# Human resource digital risk management and sustainable work–life balance in digital work environments: examining the sequential roles of mindful technology use and techno-boundary crafting

**DOI:** 10.3389/fpubh.2026.1889460

**Published:** 2026-09-01

**Authors:** Zaiba Ali, Rahila Ali, Nida Khan, Hind Saleh A. Almozeil

**Affiliations:** 1 Department of Management, College of Business Administration, Princess Nourah Bint Abdulrahman University, Riyadh, Saudi Arabia; 2 Faculty of Management, Jagran Lakecity University, Bhopal, India; 3 Department of Psychology, Fatima College of Health Sciences, Abu Dhabi, United Arab Emirates; 4 Department of Management, College of Business Administration, Princess Nourah Bint Abdulrahman University, King Khalid International Airport, Riyadh, Saudi Arabia

**Keywords:** digital work, human resource digital risk management, mindful technology use, Saudi Arabia, sequential mediation, structural equation modeling, sustainable work–life balance, techno-boundary crafting

## Abstract

**Introduction:**

As economies modernize rapidly, digital transformation has changed the nature of work itself—often faster than employees can psychologically and socially adjust to it. This study looks at how Human Resource Digital Risk Management (HRDRM) supports Sustainable Work-Life Balance (SWLB) among Saudi knowledge workers, and specifically how this happens through a two-step behavioral process: first Mindful Technology Use (MTU), then Techno-Boundary Crafting (TBC).

**Methods:**

We surveyed 665 knowledge workers across three sectors in Saudi Arabia—IT and technology services, banking and financial services, and higher education—using a quantitative cross-sectional design. The measurement and structural models were tested using Covariance-based Structural Equation Modeling (CB-SEM) in IBM AMOS, and the sequential mediation pathway was examined through bias-corrected bootstrapping with 5,000 resamples.

**Results:**

HRDRM was a significant predictor of both MTU (β = 0.382, *p* < 0.001) and SWLB directly (β = 0.338, *p* < 0.001). MTU, in turn, strongly predicted TBC (β = 0.547, *p* < 0.001), and TBC itself was positively linked to SWLB (β = 0.262, *p* < 0.001). Interestingly, HRDRM did not predict TBC directly (β = 0.036, *p* = 0.411)—its influence only emerged once MTU was factored in. This sequential indirect pathway, from HRDRM through MTU and TBC to SWLB, was statistically significant (β = 0.039, 95% CI [0.020, 0.069]), confirming the sequential mediation model.

**Discussion:**

Taken together, these findings suggest that HRDRM shapes sustainable work–life balance through two distinct routes: a direct organizational effect, and an indirect one that works through employees' own self-regulatory behaviors. The fact that Mindful Technology Use appears to be a necessary first step before employees can effectively craft techno-boundaries adds a meaningful piece to digital HRM theory. Set against the backdrop of Saudi Arabia's Vision 2030 digital transformation agenda, this study offers organizations practical insight into how they can better support employee well-being as workplaces become increasingly digitally intensive.

## 1 Introduction

The rapid digitalization of work has fundamentally altered the conditions under which employees relate to their organizations, collaborate with colleagues, and fulfill professional responsibilities—expanding flexibility, deepening connectivity, and amplifying productivity in ways that would have been structurally impossible in traditional work arrangements. Yet as evidence accumulates, a considerably more detailed picture comes into focus. Digitally intensive work environments do not merely reconfigure how work is done; they simultaneously generate a distinct and consequential set of psychosocial risks—among them techno-stress, compulsive connectivity, progressive cognitive overload, and the incremental dissolution of the psychological and temporal boundaries that once separated occupational demands from personal life. These risks are particularly pronounced in the remote and hybrid work arrangements that have become ubiquitous with digital transformation (1, 2). The assumption that digital flexibility is inherently beneficial to employee wellbeing has not withstood empirical testing; in reality, the very same technological affordances that are supposed to increase workers' autonomy and reach can equally increase the connectivity pressures, availability expectations, and attentional demands that were supposed to be reduced (3, 4). In the face of such pressures, workers are slowly shifting from passive acceptance to active, purposive engagement—adjusting, regulating, and strategically shaping their interactions with digital systems in conscious attempts to maintain work conditions that are still manageable, meaningful, and sustainable within digitally mediated settings (5).

These dynamics are especially intriguing in the case of Saudi Arabia. One of the most ambitious and concentrated experiments in deliberate national digitalization currently underway anywhere in the world, the Kingdom has invested heavily in technology-enabled work infrastructure across the public and private sectors, with digital transformation positioned as a foundational pillar of Vision 2030 and its human capital development agenda (6–8). This transformation has been unprecedented in its rapidity, creating digital work conditions more swiftly than the organizational and individual capabilities needed to manage them sustainably have developed—a gap that is especially significant in the IT, banking, and higher education sectors that have paved the path in Saudi Arabia's transition to a knowledge economy.

Existing digital work research has largely examined techno-stress and work–life balance as direct relationships, leaving largely unexplained the sequential behavioral mechanisms through which organizational digital risk governance translates into sustainable employee balance outcomes. This gap is compounded by a parallel limitation within HRM scholarship: oriented predominantly toward performance enhancement, talent acquisition, and engagement, the field has not kept pace with the distinctive psychosocial risks that digital transformation generates—techno-stress, digital overload, compulsive connectivity, and the progressive erosion of restorative boundaries between work and personal life (9). The architecture of digital work has changed profoundly; the theoretical architecture of HR has largely not. What the contemporary digital work environment demands, and what this study proposes, is a risk-oriented HRM perspective—one that treats these emerging psychosocial hazards as organizational risks requiring systematic identification, assessment, and mitigation through what we conceptualize as Human Resource Digital Risk Management. HRDRM differs from general digital wellness initiatives because it conceptualizes digitally induced psychosocial strain as an organizational risk category requiring systematic HR governance rather than isolated employee support interventions.

Although digital work and employee wellbeing have drawn growing scholarly interest, we still know surprisingly little about how organizations' digital risk management efforts actually translate into lasting work–life balance—and what role employees' own self-regulation plays in that process. This study takes up that question by tracing how Mindful Technology Use and Techno-Boundary Crafting work together, in sequence, to connect Human Resource Digital Risk Management to Sustainable Work–Life Balance.

Critically, however, this study does not position HRDRM as a direct solution to work–life balance challenges. Rather, it proposes and tests a sequential model in which HRDRM produces sustainable balance through two successive behavioral mechanisms: Mindful Technology Use (MTU) and Techno-Boundary Crafting (TBC). The theoretical logic is sequential rather than structural, reflecting a theoretically ordered process grounded in self-regulation rather than a directly observed [Reviewer Comment 2] theoretically ordered progression. Organizations that proactively manage digital work risks first cultivate employees' capacity for mindful, deliberate engagement with technology—an attentional and self-regulatory competency that is MTU. Mindful technology awareness, in turn, generates the self-knowledge, agency, and intentionality that employees need to proactively redesign the digital boundaries governing their work–personal interface—which is TBC. And it is this proactive boundary architecture that ultimately produces the durable, cumulative work–life balance that digital work environments make increasingly difficult to sustain.

The sequential mediation logic—Human Resource Digital Risk Management → Mindful Technology Use → Techno-Boundary Crafting → Sustainable Work–Life Balance—represents the study's most significant and original theoretical contribution, one that departs meaningfully from prior frameworks in the digital work and HRM literatures. To date, organizational HR practices and individual behavioral self-management strategies in digital work environments have been studied largely in isolation: HR risk management as a structural, policy-level intervention, and technology self-regulation as an individual dispositional or coping competency (9, 10). No prior study has specified the theoretically sequential behavioral sequence through which organizational HR digital risk governance is translated into individual boundary crafting capacity—and it is precisely this theoretically specified pathway that the present model maps. By demonstrating that Mindful Technology Use is theorized as a theoretical precursor to Techno-Boundary Crafting x‘and that Human Resource Digital Risk Management cultivates Mindful Technology Use as the gateway through which Sustainable Work–Life Balance is behaviorally generated—this study introduces a theoretically grounded, empirically validated architecture that is genuinely new to the literature. The concept of Human Resource Digital Risk Management itself, operationalized here as a systematic organizational process of identifying, assessing, and proactively mitigating digital psychosocial risks through HR governance, has not previously been positioned as an antecedent within a sequential behavioral mediation framework. This positioning advances the field by transforming HRDRM from a structural compliance mechanism into a theoretically coherent driver of individual behavioral self-regulation—one whose effects on Sustainable Work–Life Balance are shown to operate through two complementary pathways: a direct structural route in which HR digital risk governance reduces boundary violations at the organizational level, and an indirect theoretically specified route in which it is theorized to cultivate the mindful awareness from which proactive boundary architecture—and ultimately durable, individually owned balance—becomes possible. This dual-pathway account not only clarifies that Human Resource Digital Risk Management matters for employee wellbeing, but specifies how and through what theoretically ordered sequence it generates its effects—a level of theoretical precision that prior single-mechanism models of organizational support and work–life balance have not achieved, and that carries concrete implications for where organizations should direct their behavioral investment if they intend HR digital risk frameworks to produce sustainable outcomes rather than structural compliance alone.

With these gaps in mind, this study sets out to understand how Human Resource Digital Risk Management shapes Sustainable Work–Life Balance in workplaces where digital technology is woven into nearly everything employees do. More specifically, it asks whether organizational digital risk governance affects work–life balance directly, and also indirectly, through the sequential self-regulatory steps of Mindful Technology Use and then Techno-Boundary Crafting. The study also looks at whether Organizational Identification offers a separate, complementary psychological route linking HRDRM to Sustainable Work–Life Balance. Taken together, three questions guide the research: (1) How does Human Resource Digital Risk Management contribute to Sustainable Work–Life Balance? (2) What roles do Mindful Technology Use and Techno-Boundary Crafting play in explaining that relationship? And (3) to what extent does Organizational Identification work as an additional explanatory mechanism in this process?

## 2 Theoretical background

This study integrates four complementary theoretical frameworks to explain how Human Resource Digital Risk Management (HRDRM) generates Sustainable Work–Life Balance (SWLB) through the sequential mechanisms of Mindful Technology Use (MTU) and Techno-Boundary Crafting (TBC).

Rather than representing competing explanations, these four theoretical perspectives describe successive stages of the same self-regulatory process. Dual Process Theory explains how organizational digital risk governance shifts employees from automatic to deliberate technology engagement. Temporal Self-Regulation Theory extends this explanation by describing how deliberate regulation provides the theoretical basis for proactive boundary design. Boundary Theory explains why proactively crafted digital boundaries are essential for maintaining sustainable work–life balance, while Job Crafting Theory explains employees' active role in redesigning these boundaries. Together, these perspectives provide a coherent multilevel explanation linking organizational HR practices, employee self-regulation, proactive behavioral adaptation, and sustainable wellbeing. Collectively, these theories represent complementary stages of a single self-regulatory process rather than independent explanations of separate relationships.

### 2.1 Dual process theory

Dual Process Theory distinguishes automatic System 1 processing from deliberate System 2 processing (11, 12). In digitally intensive work environments, employees default to System 1 technology behavior—reactive checking, compulsive connectivity, and passive acceptance of digital demands. HRDRM disrupts these defaults by reducing ambient cognitive load, creating the institutional conditions in which System 2 deliberate engagement becomes organizationally accessible. MTU represents System 2 technology self-regulation; TBC represents its more advanced prospective expression, explaining why mindful awareness is a necessary precondition for proactive boundary design.

### 2.2 Temporal self-regulation theory

Temporal Self-Regulation Theory (TSRT) distinguishes immediate regulation—the real-time governance of behavior as it occurs—from prospective regulation—the advance design of conditions that pre-resolve future behavioral demands (13, 14). MTU is a form of immediate self-regulation; TBC is prospective self-regulation, wherein employees craft digital boundaries in advance to reduce future work–personal interference. TSRT predicts that prospective regulatory capacity grows from sustained immediate regulation, establishing MTU as the necessary theoretical precursor to TBC.

### 2.3 Boundary theory and job crafting theory

According to Boundary Theory, a sustainable work–life balance requires employees to be proactive in managing boundary permeability, that is, the degree to which work obligations intrude into personal life (15, 16). The fundamental nature of digital technology makes these borders transparent and hence requires proactive administration. Job Crafting Theory builds on this perspective by seeing employees as active agents who change the environment of their employment to meet their own wellbeing demands (17). In the present study context, TBC means self-initiated redesign of digital boundary architecture—and Job Crafting Theory explains why employees who are capable of intentional, thoughtful technology engagement are better positioned to execute that redesign efficiently.

To ensure theoretical precision and prevent conceptual overlap, each theory in this study is assigned a distinct and non-redundant explanatory role. Dual Process Theory explains the HRDRM → MTU relationship, capturing how organizational digital risk governance disrupts employees' automatic (System 1) technology defaults and creates the institutional conditions enabling deliberate (System 2) self-regulation. Temporal Self-Regulation Theory (TSRT) explains the MTU → TBC pathway, capturing the theoretically ordered progression from immediate, present-focused self-regulation to prospective, structurally anticipatory boundary design. Boundary Theory explains the TBC → SWLB relationship, establishing proactive management of boundary permeability as the proximal mechanism that produces sustainable work–life balance. Job Crafting Theory explains why employees who have developed mindful awareness are motivated and equipped to proactively redesign their digital boundary conditions. The distinct roles of these four frameworks are summarized in Tables 1 and A.

**Table 1:** Distinct theoretical roles in the sequential mediation model.

Theory	Explains
Dual process theory	HRDRM → MTU
Temporal self-regulation theory (TSRT)	MTU → TBC
Boundary theory	TBC → SWLB
Job crafting theory	Why employees proactively redesign boundaries after developing mindful awareness

**Table A:** Distinct theoretical roles in the sequential mediation model.

Item code	Revised item
SWLB1	I am able to maintain a consistent and healthy balance between my work responsibilities and personal life over time.
SWLB2	The boundaries between my work and personal life are sustainable and do not generate persistent or cumulative strain.
SWLB3	My current digital work arrangements allow me to engage meaningfully in both professional and personal domains.
SWLB4	I can sustain my work-life balance even when my work becomes more demanding or digitally intensive.

## 3 Literature review and hypotheses development

### 3.1 Human resource digital risk management and mindful technology use

Human Resource Digital Risk Management is the organized, systematic process of identifying, assessing and proactively managing people-related risks that arise from technology-intensive work environments, such as digital overload, techno-stress and boundary dissolution, as organizational risk categories requiring a managed response (9, 18). In the digital work context, effective HRDRM involves not only structural policy interventions—clear availability norms, digital workload limits, and recovery time protections—but also the active development of the individual behavioral competencies that employees need to manage digital demands effectively.

The link between HRDRM and mindful technology use is grounded in Dual Process Theory. In the absence of organizational support, employees in digitally intensive environments default to System 1 processing of technology demands: notifications are responded to reflexively, messages are checked compulsively, and connectivity norms are accepted passively without deliberate evaluation of their psychological costs (11, 12). HRDRM disrupts this default by creating structural conditions—reduced connectivity pressure, legitimized disengagement, and explicit organizational recognition of digital overload as a managed risk—that reduce the ambient cognitive load sustaining System 1 technology behavior and create the institutional space within which System 2, deliberate engagement becomes organizationally viable rather than professionally exceptional. When HR digital risk frameworks explicitly position compulsive connectivity as an organizational risk requiring managed response, they signal to employees that intentional technology regulation is valued and supported, substantially increasing the likelihood that employees will exercise the deliberate, awareness-based engagement with technology that Mindful Technology Use requires (19, 20). Empirically, research consistently demonstrates that organizational support for technology regulation and digital boundary management is a significant predictor of employees' capacity for deliberate, mindful digital engagement (21, 22).

Organizational support for technology regulation and boundary management creates a working climate in which employees become more attuned to their own digital behaviors and the psychological costs these behaviors carry over time. This heightened awareness—cultivated through structurally legitimized space for reflection and deliberate disengagement—facilitates the subsequent development of proactive boundary crafting behaviors, as employees who recognize the need for change are increasingly well positioned to initiate it.

*Hypothesis 1 (H1): Human Resource Digital Risk Management is positively associated with Mindful Technology Use*.

### 3.2 Mindful technology use and techno-boundary crafting

The relationship between mindful technology use and techno-boundary crafting reflects the theoretically sequential logic that Temporal Self-Regulation Theory places at the center of effective self-management over time. MTU is a form of immediate self-regulation in the TSRT sense as it is the practiced, present-focused capacity to notice, evaluate, and deliberately redirect technology behavior as it occurs, requiring the sustained exercise of System 2 attentional control (13, 19). It is not merely a strategy for reducing technology overuse; it is the sequential self-regulatory practice through which employees cultivate the self-knowledge, behavioral intentionality, and sense of personal agency over their digital behavior that increasingly ambitious self-regulatory projects require.

Techno-Boundary Crafting is, by contrast, a form of prospective self-regulation in the TSRT framework: it involves designing boundary structures—rules, routines, and digital arrangements—in advance of the situations that would otherwise demand effortful in-the-moment regulation, pre-resolving boundary decisions in ways that reduce future self-regulatory demands without requiring continuous executive control (13, 14). Applied to the digital boundary domain through Job Crafting Theory, TBC represents employees' deliberate, self-initiated redesign of the structural conditions governing how technology connects them to work demands, enacting boundaries that reflect their own preferences and recovery needs rather than organizational defaults (17, 23). Research on boundary management in digital work environments provides empirical support for this theoretically ordered sequence, demonstrating that employees who practice deliberate technology regulation are substantially more likely to engage in proactive boundary crafting (10, 24). According to sequential logic, MTU is not only related to TBC, but also a required theoretically sequential predecessor to it—the immediate self-regulatory practice that makes prospective boundary architecture cognitively and behaviorally accessible.

Although boundary crafting and mindful technology use are conceptually related, they operate at fundamentally different stages of the self-regulatory process and cannot be treated as interchangeable or parallel mechanisms. Mindful Technology Use reflects present-focused awareness and the intentional, real-time regulation of digital behavior—the practiced capacity to notice, evaluate, and redirect technology engagement as it unfolds. Techno-Boundary Crafting, by contrast, represents proactive redesign of the structural conditions governing future technology use, enacted in advance of the situations that would otherwise demand effortful in-the-moment regulation. Employees simply cannot intentionally redesign the digital boundaries structuring their work–personal interface before they have first developed awareness of their own technology habits, the intrusive patterns those habits generate, and the attentional and psychological costs those patterns carry—any more than one can architect a solution to a problem one has not yet diagnosed. Consistent with Temporal Self-Regulation Theory (13, 14), the mindfulness literature (19), and Job Crafting Theory's proposition that employees redesign their work conditions only after recognizing a need for redesign (17), awareness-based self-regulation necessarily precedes prospective behavioral redesign. MTU is therefore a cognitive precondition for TBC, not a parallel alternative to it: it provides the self-knowledge, intentionality, and sense of personal agency that make boundary crafting not merely possible, but purposeful.

*Hypothesis 2 (H2): Mindful Technology Use is positively associated with Techno-Boundary Crafting*.

### 3.3 Techno-boundary crafting and sustainable work–life balance

Techno-Boundary Crafting—the proactive, self-initiated process through which employees design and redesign the structural conditions governing how digital technology connects them to work demands—is theoretically positioned, within both Boundary Theory and Job Crafting Theory, as the proximate behavioral mechanism sustaining work–life balance in digital work environments. Unlike purely attentional mechanisms such as MTU, which improve the quality of technology engagement without altering the structural parameters governing its frequency and intrusion, TBC operates on those structural parameters directly, reducing the number, intensity, and unpredictability of work–personal boundary violations before they occur (16, 23). In Boundary Theory terms, TBC enables employees to actively manage the permeability and flexibility of their work–personal boundaries in ways that align with their individual recovery needs rather than organizational connectivity defaults—converting the inherently permeable digital boundary landscape into one shaped by deliberate personal agency (15, 16).

The wellbeing benefits of proactive boundary crafting are both structural and psychological. From a structural perspective, employees who develop digital boundaries experience fewer intrusions after work hours, more predictable patterns of connectivity, and greater consistency between their behavior with technology and their personal needs for recovery—all of which lessen the cumulative cognitive and emotional strain that the dissolution of boundaries produces over time (21, 24). Psychologically, the act of crafting one's own boundaries—rather than accepting organizational defaults—enhances employees' sense of personal agency over their work–life interface, which is a resource that sustains wellbeing independently of the structural changes it produces (17). Critically, from a TSRT perspective, TBC's prospective architecture converts what would otherwise be a continuous self-regulatory burden into a one-time design investment, making sustainable balance achievable without depleting the executive control resources that moment-to-moment boundary defense demands (13). Empirically, proactive digital boundary management is consistently associated with lower techno-stress, reduced work–life conflict, and more sustainable balance outcomes (10, 25).

*Hypothesis 3 (H3): Techno-Boundary Crafting is positively associated with Sustainable Work–Life Balance*.

### 3.4 Human resource digital risk management and sustainable work–life balance

The direct relationship between HRDRM and SWLB is grounded in Boundary Theory's proposition that sustainable balance depends on the governance of boundary permeability conditions—and that when organizations institutionally manage these conditions, they directly influence employee wellbeing outcomes independent of individual behavioral strategies (15, 16). At the structural level, HRDRM encompasses proactive organizational interventions—regulated digital availability norms, protected recovery time, and monitored workload limits—that directly reduce the frequency and intensity of work–personal boundary violations (9, 18). By managing the digital permeability of work–personal boundaries at the organizational level, HRDRM reduces cross-domain intrusions structurally, producing balance improvements that do not require employees to have fully developed the behavioral self-management competencies of mindful technology use or proactive boundary crafting. This structural dimension of HRDRM's impact represents a direct organizational-to-individual pathway to sustainable balance that complements the sequential behavioral chain.

From a Dual Process Theory perspective, HRDRM's direct effect on SWLB reflects an organizational-level reduction in the System 1 cognitive load that generates cumulative boundary erosion over time (11, 12). When HR digital risk frameworks actively manage connectivity expectations and protect personal time from work intrusion, employees experience reduced cross-domain interference not because they have individually managed these boundaries, but because the organizational architecture has structurally reduced the digital demands that erode them. Temporal Self-Regulation Theory further supports this pathway: organizations that govern digital availability expectations effectively reduce the prospective self-regulatory demands employees would otherwise need to manage individually, producing direct improvements in balance sustainability (13). Empirically, organizational support for digital boundary management consistently demonstrates significant direct effects on work–life balance outcomes (10, 21, 24), establishing the direct HRDRM–SWLB relationship as both theoretically coherent and empirically grounded.

*Hypothesis 4 (H4): Human Resource Digital Risk Management is positively associated with Sustainable Work–Life Balance*.

### 3.5 Sequential mediation: MTU and TBC in the HRDRM–SWLB relationship

The full sequential mediation hypothesis integrates H1, H2, and H3 into a unified theoretically sequential account of how organizational HR digital risk practices generate sustainable work–life balance through successive individual behavioral mechanisms, each grounded in the theoretical frameworks articulated in Section 2. HRDRM initiates the process by creating organizational conditions—reduced cognitive load, institutional legitimization of deliberate disengagement, and structural support for technology regulation—that disrupt System 1 technology defaults and enable System 2 engagement, thereby cultivating the foundation for Mindful Technology Use (11, 12). MTU, through the immediate self-regulatory practice it represents, is theorized to foster the self-knowledge, intentionality, and behavioral self-efficacy that TSRT identifies as the enabling conditions for prospective self-management—generating the capacity for proactive boundary design that TBC represents (13, 14). TBC, finally, produces the prospective boundary architecture through which sustainable work–life balance is maintained without ongoing self-regulatory strain, as employees actively craft the structural conditions governing their digital work–life interface in ways that align with their personal recovery and wellbeing needs (16, 17).

This sequential logic implies that HRDRM's influence on SWLB operates through two complementary pathways: a direct structural pathway (H4), in which organizational digital risk governance reduces boundary violations at source, and an indirect sequential pathway (H5), in which HRDRM first cultivates mindful technology awareness, which in turn enables the proactive boundary design that sustains long-term balance. The non-significant direct path from HRDRM to TBC suggests that mindful technology use may represent an important intervening mechanism through which organizational digital risk governance contributes to employees' boundary crafting capacity. It further implies that TBC is not a parallel behavioral mechanism operating independently of MTU, but is sequentially enabled by it through the cognitive and self-regulatory development that present-focused mindful engagement is theorized to support. The sequential model, grounded in Dual Process Theory, Temporal Self-Regulation Theory, Boundary Theory, and Job Crafting Theory, offers a more theoretically precise and practically informative account of the HRDRM–SWLB relationship than either parallel or single-mediator models can provide, and advances the digital work literature by specifying the theoretically ordered sequence through which organizational HR digital risk practices generate sustainable individual wellbeing outcomes.

*Hypothesis 5 (H5): Mindful Technology Use and Techno-Boundary Crafting sequentially mediate the relationship between Human Resource Digital Risk Management and Sustainable Work–Life Balance*.

## 4 Methodology

### 4.1 Research design and context

A quantitative cross-sectional survey design was applied to assess the proposed sequential mediation model. For both theoretical and practical grounds Saudi Arabia was picked as the context for the study. The Kingdom, one of the world's most rapidly digitalizing economies, has prioritized digital transformation as a key pillar of Vision 2030 and the Human Capability Development Program and has invested heavily in technology-enabled infrastructure, artificial intelligence, digital governance, and workforce modernization initiatives across public and private sectors (7, 8, 26, 27). These advances have greatly shaped organizational practices and employee work experiences, especially in technologically intensive industries including as information technology, banking, and higher education (6). Therefore, Saudi Arabia provides a particularly relevant context to explore the influence of Human Resource Digital Risk Management, mindful technology regulation and proactive boundary management on Sustainable Work-Life Balance in fast changing digital work settings.

### 4.2 Sample and data collection

The respondents were knowledge workers from three main industries in Saudi Arabia that are technologically intensive, namely information technology and technology services, banking and financial services, and higher education. A purposive sample approach was adopted with an aim to ensure that all respondents worked in technology mediated work settings and had adequate organizational tenure to effectively assess their employer's digital risk management procedures and their technology-related behavioral patterns. To preserve contextual validity and response quality, only personnel with at least 12 months organizational tenure were eligible for inclusion. Data were collected through a structured online questionnaire, distributed using Google Forms, and sent through professional email networks, intra-organizational connections and digitally mediated communication channels within the sectors of interest. Participants were informed about the academic purpose of the study, assured of confidentiality and anonymity, and provided informed consent prior to participation. All 715 initial responses received were thoroughly reviewed for incomplete responses, missing data, duplicate submissions and response inconsistencies. This led to retaining 665 complete and valid responses for analysis, a sample size well above the minimum thresholds advocated for covariance-based Structural Equation Modeling and suitable for the estimation demands of the suggested sequential mediation model (28, 29).

### 4.3 Measures

#### 4.3.1 Measurement scales

*All items rated on a 5-point Likert scale: 1* = *Strongly Disagree to 5* = *Strongly Agree*.

##### 4.3.1.1 Scale 1: human resource digital risk management (HRDRM)

*Theoretical basis:* HR digital risk management is conceptualized as the systematic organizational process of identifying, assessing, and mitigating people-related risks arising from digitally mediated work environments, including workforce vulnerabilities, digital fatigue, and capability erosion (9, 18).

Although HRDRM originates at the organizational level as a system of policies, structures, and governance mechanisms, the construct is operationalized and measured in this study at the individual level of analysis, capturing employees' perceptions of how their organization identifies, assesses, and mitigates digital work risks. This approach is theoretically grounded and empirically well-precedented. Consistent with HR attribution theory (41) and perceived organizational support research (42), it is employees' perceived experience of HR practices—not the objective existence of those policies—that shapes individual attitudes and behaviors. Two individuals working in the same organization may evaluate the same HR policy quite differently depending on how visible, meaningful, and consistently enacted they perceive it to be. Accordingly, the theoretically and practically relevant construct in this model is Employees' Perceived Human Resource Digital Risk Management Practices, and the scale items are designed to capture individual perceptions of organizational digital risk management rather than to describe organizational policy content directly. This individual-level conceptualization is both appropriate for the behavioral outcomes under study and consistent with the broader tradition of measuring HR practices through employee perception in HRM research.

Employees' Perceived Human Resource Digital Risk Management Practices were measured using a four-item scale developed to capture organizational efforts to identify, regulate, and mitigate people-related digital work risks. The scale was designed to reflect the multidimensional nature of digital risk governance as experienced by employees in digitally intensive work environments. Consistent with HRM and organizational behavior research, several items intentionally capture closely related manifestations of digital risk management rather than isolated operational activities. For instance, digital overload, techno-stress, and excessive connectivity pressures frequently co-occur within digitally intensive work systems and are often addressed through the same organizational interventions. Accordingly, the scale was designed to capture employees' holistic perceptions of organizational digital risk governance rather than isolated manifestations of individual digital work risks.

**Adapted from:** Armstrong and Taylor (9) HR practice effectiveness framework; Nocco and Stulz (18) enterprise risk management dimensions. The measurement items for Human Resource Digital Risk Management (HRDRM) and Sustainable Work-Life Balance (SWLB) are presented in Table A1.

**Table A1:** Measurement items for Human Resource Digital Risk Management (HRDRM) and Sustainable Work–Life Balance (SWLB).

Item code	Revised item
HRDRM1	My organization actively identifies and addresses employee well-being risks associated with digitally intensive work.
HRDRM2	HR policies in my organization help reduce digital overload, techno-stress, and excessive connectivity pressures.
HRDRM3	My organization promotes healthy digital work practices that protect employees' recovery time and psychological well-being.
HRDRM4	HR practices in my organization are regularly adapted to support employee well-being in evolving digital work environments.

##### 4.3.1.2 Scale 2: sustainable work-life balance (SWLB)

*Theoretical basis:* Sustainable work-life balance is conceptualized as the ongoing, durable capacity of employees to maintain equitable and satisfying engagement across work and personal domains over time, without accumulating chronic strain or sacrificing either sphere (30, 31).

**Adapted from:** Haar (30) 4-item Work-Life Balance Scale (*International Journal of Human Resource Management*); Fisher et al. (31) Work/Nonwork Interference and Enhancement Scale (*Journal of Occupational Health Psychology*).

##### 4.3.1.3 Scale 3: mindful technology use (MTU)

*Theoretical basis:* Mindful technology use is defined as the deliberate, awareness-based engagement with digital technologies in which employees consciously regulate their technology behavior, monitor its psychological effects, and make intentional choices about when, how, and why they use digital tools (19, 32).

**Adapted from:** Brown and Ryan (19) Mindful Attention Awareness Scale (MAAS), adapted for digital technology contexts (*Journal of Personality and Social Psychology*); Rosen et al. (32) Media and Technology Usage and Attitudes Scale (MTUAS) (*Computers in Human Behavior*). The measurement items used to assess Mindful Technology Use (MTU) are presented in Table A2.

**Table A2:** Measurement items for Mindful Technology Use (MTU).

Item code	Revised item
MTU1	I consciously decide when and how to use digital technologies during my workday rather than responding to them automatically or out of habit.
MTU2	I am aware of how my technology use affects my concentration, stress levels, and overall psychological well-being.
MTU3	I deliberately limit my engagement with digital devices during personal time to protect my rest and psychological recovery.
MTU4	I engage with digital tools with a clear and intentional purpose, avoiding habitual or compulsive checking behaviors.

##### 4.3.1.3 Scale 4: techno-boundary crafting (TBC)

*Theoretical basis:* Techno-boundary crafting is conceptualized as the proactive, self-initiated process through which employees actively shape, negotiate, and redesign the digital boundaries between their work and personal lives—going beyond passive compliance with organizational technology norms to craft personalized boundary structures that reflect their values, preferences, and recovery needs (17, 23, 24).

**Adapted from:** Wrzesniewski and Dutton (17) job crafting framework (*Academy of Management Review*); Kossek et al. (23) work-nonwork boundary management profiles (*Journal of Vocational Behavior*); Park et al. (24) ICT boundary management scale (*Journal of Organizational Behavior*). The measurement items used to assess Techno-Boundary Crafting (TBC) are presented in Table A3.

**Table A3:** Measurement items for Techno-Boundary Crafting (TBC).

Item code	Revised item
TBC1	I proactively set personal rules about when and how work-related technology can enter my personal time.
TBC2	I intentionally shape the digital boundaries between my work and personal spaces to align with my own preferences and well-being needs.
TBC3	I actively modify how digital technologies connect me to work demands in order to protect my personal recovery time.
TBC4	I craft my own digital boundaries rather than simply accepting or complying with organizational technology expectations.

### 4.4 Analytical approach

Data were analyzed using covariance-based SEM in IBM AMOS, adopting the Anderson and Gerbing (33) two-step method to SEM: first, measurement model evaluation, and second, structural model testing. Reliability was evaluated by Cronbach's alpha (α ≥ 0.70) and Composite Reliability (CR ≥ 0.70). For convergent validity, standardized factor loadings (≥ 0.60) and Average Variance Extracted (AVE > 0.50; 34) were examined. Discriminant validity was examined using the Fornell–Larcker criterion and the HTMT ratios (< 0.85; 35). The model fit was assessed in terms of χ^2^/df, CFI, TLI, IFI, NFI, and RMSEA (29, 36). Finally, to test the sequential mediation hypothesis (H5), we used bias-corrected bootstrapping (5,000 resamples) to calculate 95% confidence intervals for the indirect effect of HRDRM on SWLB through MTU and then TBC (37). Common method bias was evaluated using Harman's one-factor test with procedural remedies implemented during data collection (38).

## 5 Results

**Figure 1:**
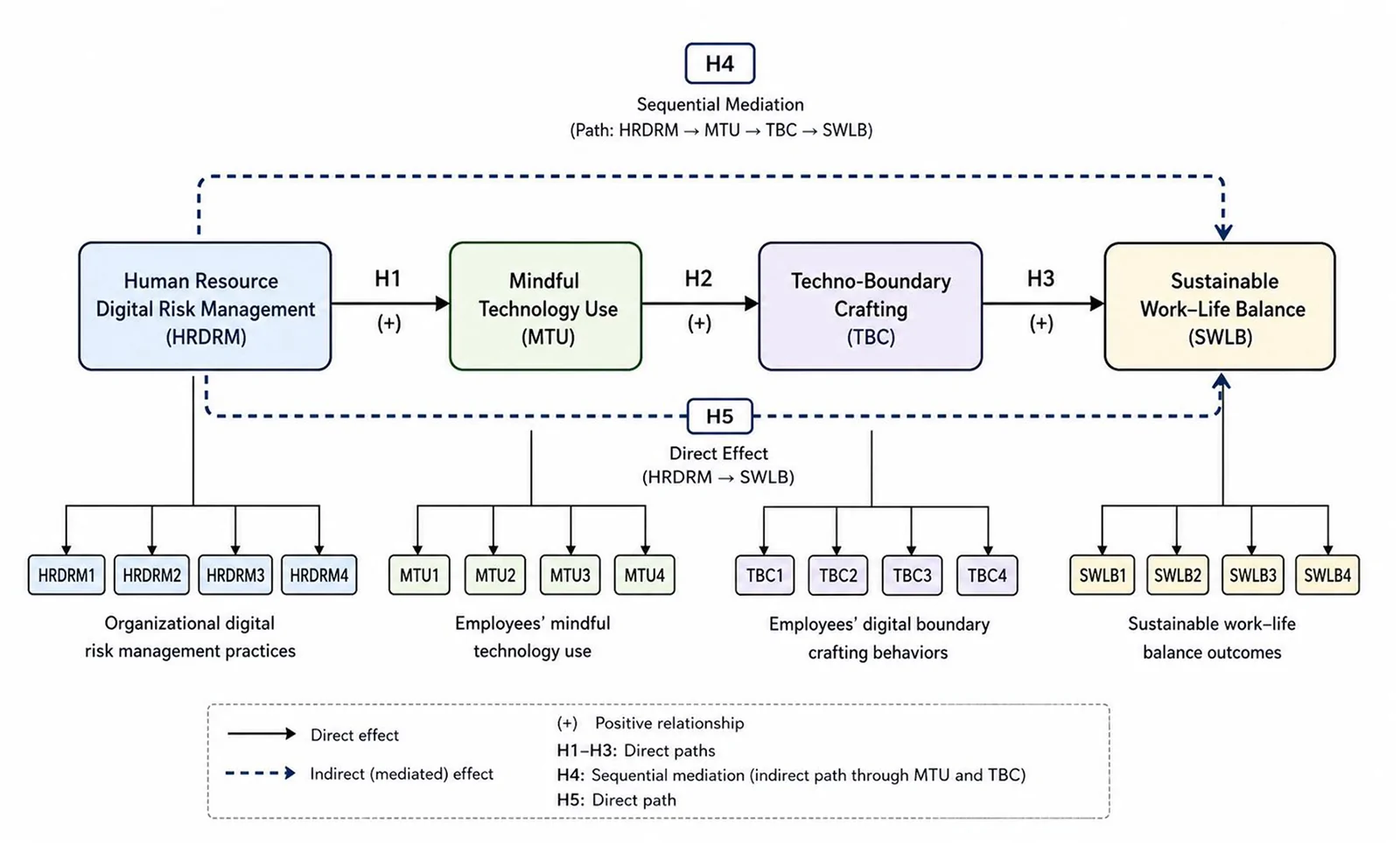
Conceptual framework: sequential mediation model.

Figure 1 displays the conceptual framework of the proposed partial sequential mediation model. The exogenous variable, Human Resource Digital Risk Management (HRDRM), is expected to influence Sustainable Work–Life Balance (SWLB; H4) directly and indirectly, through a sequential behavioral path. Specifically, HRDRM positively predicts Mindful Technology Use (MTU; H1) which in turn positively predicts Techno-Boundary Crafting (TBC; H2). H3: TBC has a beneficial impact on SWLB. Also, the model predicts the sequential mediation of MTU and TBC between HRDRM and SWLB (H5). This yields a dual path model of organizational digital risk governance toward sustainable balance, *via* direct structural support and gradual behavioral self-regulation.

### 5.1 Sample characteristics

Table 2 presents the demographic and organizational profile of the 665 respondents. The sample was drawn from three digitally intensive sectors in Saudi Arabia: IT and technology services constituted the largest proportion (39.7%, *n* = 264), followed by higher education and EdTech (34.0%, *n* = 226) and banking and financial services including FinTech and digital banking (26.3%, *n* = 175). This sectoral composition reflects a predominantly knowledge-worker, technology-engaged sample, which is well-suited to the study's examination of digital work demands, technology self-regulation, and boundary management behaviors.

**Table 2:** Demographic profile of respondents (*N* = 665).

Variable	Category	*n*	Percentage (%)
Sector	IT/Technology Services	264	39.7
	Banking and Financial Services (FinTech/Digital Banking)	175	26.3
	Higher Education/EdTech (Universities and Online Learning)	226	34.0
Total		665	100.0
Organizational tenure	More than 48 months	380	57.1
	24–48 months	190	28.6
	12–24 months	95	14.3
Total		665	100.0
Gender	Male	359	54.0
	Female	306	46.0
Total		665	100.0

Respondents were predominantly experienced employees: 57.1% reported organizational tenure exceeding 48 months, with a further 28.6% having 24–48 months of experience, and 14.3% between 12 and 24 months. This suggests that the majority of participants possessed sufficient organizational exposure to evaluate their employers' HR digital risk management practices meaningfully and to have developed established patterns of technology use and boundary management over time. Gender representation was broadly balanced, with male respondents comprising 54.0% (*n* = 359) and female respondents 46.0% (*n* = 306) of the sample.

### 5.2 Descriptive statistics and normality assessment

Descriptive statistics for all 16 measurement items are reported in Table 3. Human Resource Digital Risk Management (HRDRM) item means ranged from 3.39 (HRDRM3) to 3.60 (HRDRM2), placing perceptions of organizational digital risk management modestly above the scale midpoint. Sustainable Work–Life Balance (SWLB) means ranging from 3.02 (SWLB2) to 3.18 (SWLB4), reflecting a moderately low level of perceived work–life balance sustainability among respondents in Saudi Arabia's digitally intensive sectors.

**Table 3:** Descriptive statistics of measurement items (*N* = 665).

Const.	Item	N	Min	Max	Mean	SD	Skew.	Kurt.
HRDRM	HRDRM1—Identifies digital work risks systematically	665	1	5	3.55	1.47	0.013	−0.875
	HRDRM2—Proactively addresses burnout and digital fatigue	665	1	5	3.60	1.55	0.075	−0.985
	HRDRM3—Contingency plans for digital work pressure	665	1	5	3.39	1.64	0.173	−1.049
	HRDRM4—HR practices reviewed for emerging digital risks	665	1	5	3.44	1.47	0.280	−0.488
MTU	MTU1—Consciously decides when to use digital technology	665	1	5	2.86	1.17	0.251	−0.811
	MTU2—Aware of technology's psychological effects	665	1	5	3.00	1.28	0.090	−1.137
	MTU3—Deliberately limits devices during personal time	665	1	5	2.86	1.19	0.208	−0.984
	MTU4—Engages with purpose; avoids compulsive checking	665	1	5	2.72	1.22	0.413	−0.845
TBC	TBC1—Proactively sets rules for technology in personal time	665	1	5	2.95	1.17	0.105	−0.852
	TBC2—Intentionally shapes digital work-personal boundaries	665	1	5	2.74	1.09	0.477	−0.356
	TBC3—Actively modifies digital connections for recovery	665	1	5	2.84	1.08	0.346	−0.495
	TBC4—Crafts own boundaries beyond organizational norms	665	1	5	2.89	1.13	0.274	−0.647
SWLB	SWLB1—Consistent healthy balance maintained over time	665	1	5	3.05	1.48	0.533	−0.468
	SWLB2—Boundaries sustainable without chronic strain	665	1	5	3.02	1.30	0.625	0.293
	SWLB3—Meaningful engagement across both work and personal domains	665	1	5	3.05	1.37	0.567	0.085
	SWLB4—Sustainable even under intensified digital demands	665	1	5	3.18	1.45	0.612	−0.002

All skewness and kurtosis values fall within the acceptable thresholds of ±2 and ±7 respectively, confirming distributional suitability for maximum likelihood estimation (28, 29).

Mindful Technology Use (MTU) means ranged from 2.72 (MTU4) to 3.00 (MTU2), suggesting that deliberate, awareness-based technology regulation remains a developing competency in this sample. Techno-Boundary Crafting (TBC) means ranged from 2.74 (TBC2) to 2.95 (TBC1), indicating similarly moderate engagement with proactive digital boundary crafting behaviors. Together, the moderately low mean scores across MTU and TBC are theoretically informative: they suggest that the behavioral self-management capacities targeted by the sequential mediation model are not yet widely or consistently practiced in Saudi Arabia's rapidly digitalizing work environments, underscoring both the relevance of the research question and the practical urgency of the study's findings.

Normality was assessed through inspection of skewness and kurtosis values, as required for maximum likelihood estimation in covariance-based SEM (29). All skewness values ranged from 0.013 (HRDRM1) to 0.625 (SWLB2), and kurtosis values from −1.137 (MTU2) to 0.293 (SWLB2). All values fell well within the conventionally accepted thresholds of ±2 for skewness and ±7 for kurtosis (28), confirming that univariate normality assumptions were not seriously violated and that the data were suitable for subsequent confirmatory factor analysis and structural model estimation.

### 5.3 Common method bias assessment

Harman's single-factor test was subsequently employed as a *post hoc* statistical examination of common method variance. An unrotated principal component analysis was conducted on all 16 measurement items, and the results, presented in Table 4, reveal a favorable pattern. Rather than yielding a single dominant factor accounting for a majority of variance—the pattern conventionally taken to indicate problematic common method bias—the analysis identified four distinct components with eigenvalues exceeding 1.0, jointly explaining 68.735% of the total variance. Crucially, the first component accounted for only 32.404% of total variance, substantially below the 50% threshold commonly used to signal concerning levels of common method bias (38). Moreover, the even distribution of explained variance across four components, each of which corresponds conceptually to one of the study's latent constructs, provides reassuring evidence that respondents meaningfully differentiated among the measured variables rather than responding through a generalized, method-driven tendency.

**Table 4:** Total variance explained (Common Method Bias Test).

Component	Construct	Eigenvalue	% of Variance	Cumulative %
1	Human resource digital risk management (HRDRM)	5.185	32.404	32.404
2	Mindful technology use (MTU)	2.542	15.889	48.293
3	Sustainable work–life balance (SWLB)	1.972	12.327	60.619
4	Techno-boundary crafting (TBC)	1.299	8.116	68.735

Extraction method: Principal Component Analysis (unrotated solution). Four components with eigenvalues greater than 1 were extracted, jointly explaining 68.735% of the total variance.

Taken together, the procedural precautions implemented during data collection and the statistical evidence derived from Harman's single-factor test converge to indicate that common method bias is unlikely to pose a material threat to the validity of the study's substantive findings. The data were accordingly deemed appropriate for advancing to measurement and structural model analyses.

### 5.4 Correlation analysis

Table 5 displays the Pearson correlation coefficients for the four constructs studied. All correlations were statistically significant at the *p* < 0.01 level and collectively suggested a pattern of positive, theoretically consistent relationships that provide preliminary empirical support for the proposed sequential mediation model. Human Resource Digital Risk Management (HRDRM) was found to have moderate but significant positive associations with Mindful Technology Use (*r* = 0.35, *p* < 0.01), Sustainable Work–Life Balance (*r* = 0.37, *p* < 0.01), and Techno-Boundary Crafting (*r* = 0.21, *p* < 0.01). This implies that employees who perceive their organizations as actively involved in digital risk management are more likely to report higher levels of deliberate technology self-regulation, intentional boundary crafting, and perceived balance sustainability. For its part, Mindful Technology Use had significant positive correlations with Sustainable Work–Life Balance (*r* = 0.36, *p* < 0.01) and Techno-Boundary Crafting (*r* = 0.21, *p* < 0.01), indicating that, although awareness-based technology use and proactive boundary design are empirically distinct, they have a significant behavioral commonality that can be tied to volitional self-regulation. The highest correlation in the matrix was between Techno-Boundary Crafting and Sustainable Work–Life Balance (*r* = 0.55, *p* < 0.01), an interesting, statistically and theoretically, finding that points us to proactive digital boundary management as the construct most proximal and directly associated with durable work–life balance outcomes—consistent with boundary theory views that suggest that volitional role-boundary control is a primary determinant of perceived balance sustainability over a distal or incidental correlate (15, 39). However, importantly, the inter-construct correlations were all below the recommended threshold of 0.85, indicating appropriate discriminant validity and no multicollinearity issues that could otherwise jeopardize the accuracy of later structural parameter estimates (28).

**Table 5:** Means, standard deviations, and correlations among study constructs.

Construct	Mean	SD	1	2	3	4
1. Human resource digital risk management (HRDRM)	3.42	1.31	—			
2. Mindful technology use (MTU)	3.18	1.14	0.35^**^	—		
3. Sustainable work–life balance (SWLB)	3.06	1.27	0.37^**^	0.36^**^	—	
4. Techno-boundary crafting (TBC)	2.91	1.09	0.21^**^	0.21^**^	0.55^**^	—

*N* = 665. Values represent Pearson correlation coefficients. *p* < 0.01. The symbol ^**^ indicates that the correlation is statistically significant at the 0.01 level (two-tailed) (*p* < 0.01).

### 5.5 Measurement model

**Figure 2:**
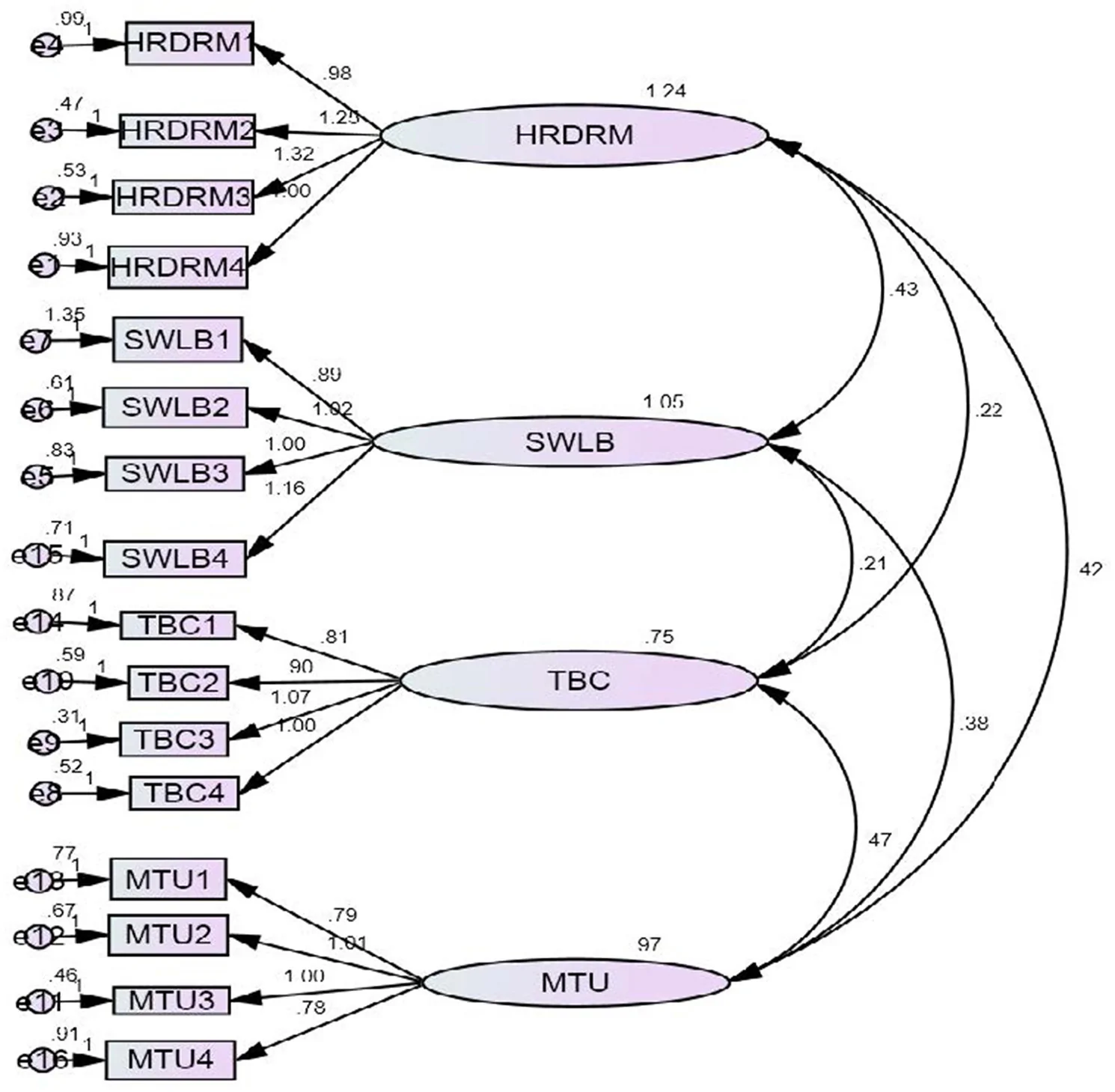
Presents the confirmatory factor analysis (CFA) measurement model for the four latent constructs: human resource digital risk management (HRDRM), mindful technology use (MTU), techno-boundary crafting (TBC), and sustainable work–life balance (SWLB).

Confirmatory Factor Analysis confirmed acceptable model fit (Table 6): χ^2^ (100) = 429.629, χ^2^/df = 4.296, NFI = 0.917, RFI = 0.900, IFI = 0.935, TLI = 0.922, CFI = 0.935, RMSEA = 0.071 [90% CI: 0.064, 0.078]. While the χ^2^/df exceeded the conservative threshold of 3.0, this is expected with N = 662, where chi-square is particularly sensitive to minor model misspecifications; the incremental fit indices collectively support adequate model fit (28, 29). All standardized factor loadings exceeded 0.60 (Table 7), confirming acceptable item reliability. AVE values exceeded 0.50 and CR values exceeded 0.70 for all constructs, establishing convergent validity. Discriminant validity was confirmed *via* Fornell–Larcker criterion and HTMT ratios below 0.85 (35). Harman's single-factor test did not indicate serious common method bias concerns (Figure 2; Tables 8–11).

**Table 6:** Measurement model fit indices.

χ^2^/df (CMIN/DF)	GFI	AGFI	NFI	IFI	TLI	CFI	RMSEA
3.615	0.936	0.912	0.931	0.949	0.938	0.949	0.063

The measurement model demonstrated satisfactory goodness-of-fit across all major fit indices, indicating acceptable model adequacy for Confirmatory Factor Analysis (CFA).

**Table 7:** Measurement model: factor loadings, AVE, CR, and reliability.

Construct	Item	Std. Loading	AVE	CR	Cronbach's α
HRDRM	HRDRM1	0.740			
	HRDRM2	0.893	0.681	0.894	0.786
	HRDRM3	0.901			
	HRDRM4	0.753			
MTU	MTU1	0.658			
	MTU2	0.775	0.526	0.814	0.822
	MTU3	0.826			
	MTU4	0.624			
TBC	TBC1	0.605			
	TBC2	0.709	0.547	0.826	0.850
	TBC3	0.851			
	TBC4	0.771			
SWLB	SWLB1	0.625			
	SWLB2	0.796	0.565	0.837	0.857
	SWLB3	0.761			
	SWLB4	0.809			

AVE, average variance extracted; CR, composite reliability. All loadings ≥ 0.60; AVE ≥ 0.50; CR ≥ 0.70 (34). *R*^2^ for constructs from SEM output.

**Table 8:** Explained variance (*R*^2^) of endogenous constructs.

Construct	*R* ^2^
MTU	0.150
TBC	0.306
SWLB	0.170

*R*^2^ values represent the proportion of variance explained in each endogenous construct by its predictor variables in the structural model.

**Table 9:** Fornell–Larcker criterion for discriminant validity.

Construct	HRDRM	MTU	SWLB	TBC
HRDRM	**0.823**			
MTU	0.382	**0.722**		
SWLB	0.374	0.379	**0.747**	
TBC	0.228	0.548	0.236	**0.737**

Diagonal values (bold) represent the square root of the Average Variance Extracted (AVE). Off-diagonal values represent inter-construct correlations.

**Table 10:** HTMT criterion for discriminant validity.

Construct	HRDRM	MTU	SWLB	TBC
HRDRM	—			
MTU	0.451	—		
SWLB	0.433	0.471	—	
TBC	0.281	0.642	0.318	—

All HTMT values were below the recommended threshold of 0.85, indicating satisfactory discriminant validity and confirming that the constructs are empirically distinct.

**Table 11:** Structural model fit indices.

χ^2^/df (CMIN/DF)	NFI	RFI	IFI	TLI	CFI	RMSEA
3.777	0.928	0.912	0.946	0.934	0.945	0.065

χ^2^/df = chi-square divided by degrees of freedom; NFI, normed fit index; RFI, relative fit index; IFI, incremental fit index; TLI, Tucker–Lewis index; CFI, comparative fit index; RMSEA, root mean square error of approximation.

### 5.6 Structural model and direct effects

**Figure 3:**
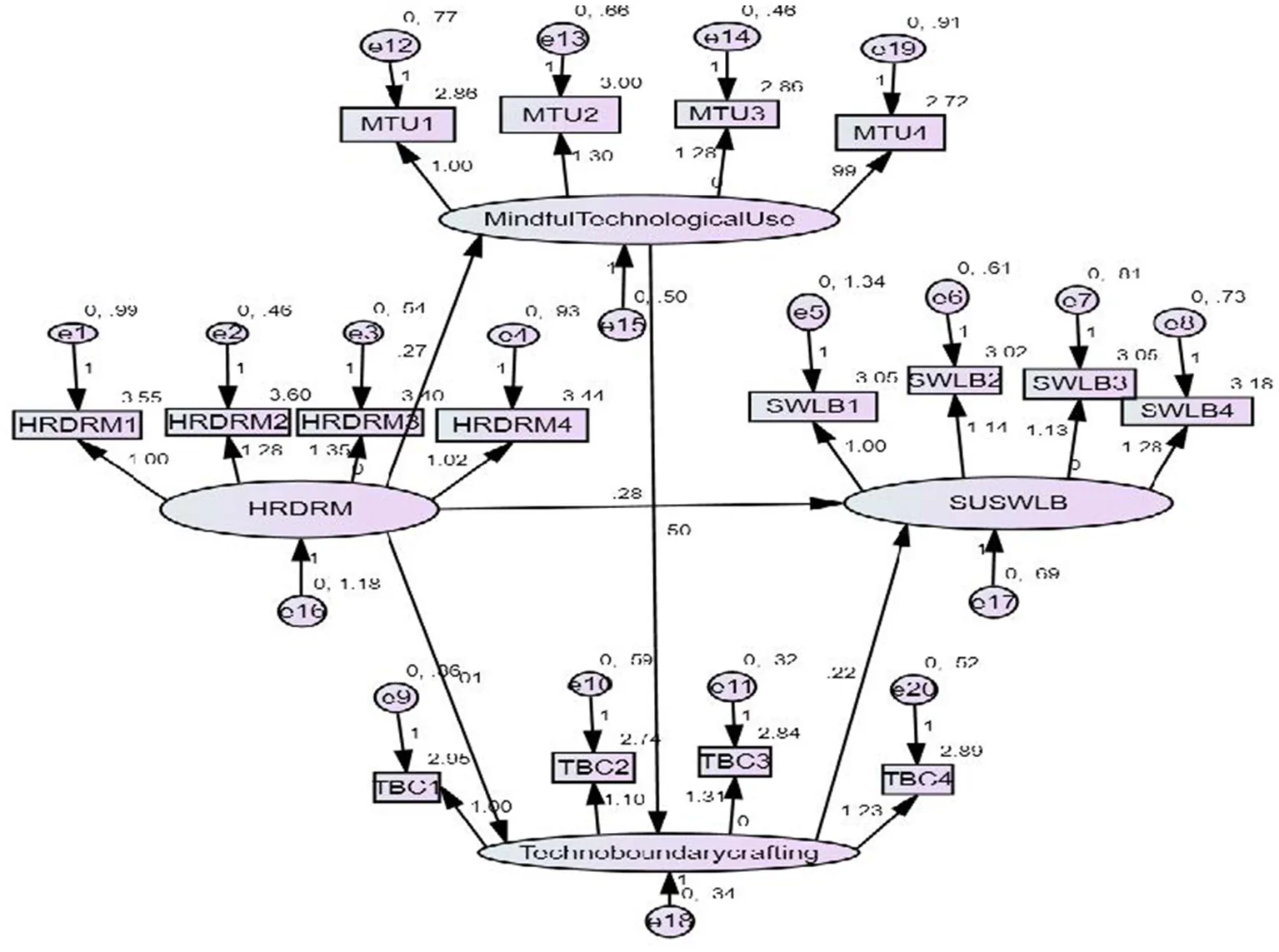
Structural equation model of human resource digital risk management, mindful technology use, techno-boundary crafting, and sustainable work–life balance.

The structural model tested the sequential mediation pathway HRDRM → MTU → TBC → SWLB. Table 12 presents all path coefficients. HRDRM significantly and positively predicted MTU (H1: Std *β* = 0.382, *β* = 0.270, CR = 8.042, *p* < 0.001), confirming that organizational HR digital risk management practices create the enabling conditions for mindful technology engagement. MTU exerted a strong and significant effect on TBC (H2: Std *β* = 0.547, *β* = 0.501, CR = 9.174, *p* < 0.001), representing the strongest path in the model and establishing mindful awareness as the primary driver of proactive boundary crafting. TBC, in turn, significantly predicted SWLB (H3: Std *β* = 0.262, *β* = 0.343, CR = 5.409, *p* < 0.001), confirming proactive boundary design as the proximate behavioral mechanism producing sustainable balance outcomes (Figure 3).

**Table 12:** Structural model path estimates.

H	Path	*β* (Unstd.)	SE	CR	*p*	Std. *β*	Result
H1	HRDRM → MTU	0.270	0.034	8.042	^***^	0.382	Supported
H2	MTU → TBC	0.501	0.055	9.174	^***^	0.547	Supported
H3	TBC → SWLB	0.343	0.063	5.409	^***^	0.262	Supported
H4	HRDRM → SWLB	0.281	0.049	5.732	^***^	0.338	Supported
—	HRDRM → TBC	0.023	0.028	0.823	0.411	0.036	Not Significant

*β*, unstandardized path coefficient; SE, standard error; CR, critical ratio; Std. *β*, standardized path coefficient. ^***^p < 0.001. HRDRM, human resource digital risk management; MTU, mindful technology use; TBC, techno-boundary crafting; SWLB, sustainable work–life balance.

Critically, the direct effect of HRDRM on TBC was non-significant (Std *β* = 0.036, CR = 0.823, *p* = 0.411), confirming that the findings support the theorized role of mindful technology awareness as the intervening mechanism through which boundary crafting capacity operates—rather than operating directly from organizational policy. Importantly, the direct effect of HRDRM on SWLB was significant (H4: Std *β* = 0.338, *β* = 0.281, SE = 0.049, CR = 5.732, *p* < 0.001), establishing that HRDRM produces balance improvements through both a direct structural pathway and the sequential behavioral chain. Together, these results support a partial sequential mediation model in which organizational HR digital risk management generates sustainable balance through two complementary and theoretically distinct mechanisms (Table 12).

### 5.7 Sequential mediation analysis

The sequential mediation hypothesis (H5) was tested using bias-corrected bootstrapping with 5,000 resamples. The sequential indirect effect of HRDRM on SWLB through MTU and then TBC was *β* = 0.039 [95% CI: 0.020, 0.069]. Because this confidence interval does not include zero, the sequential indirect effect is statistically significant, confirming H5. Alongside the significant direct HRDRM → SWLB path (H4: Std *β* = 0.338, *p* < 0.001), these results establish a partial sequential mediation structure: HRDRM influences sustainable balance through both a direct structural pathway and the sequential self-regulatory development of mindful technology use and proactive boundary crafting.

Table 13 presents the mediation results. These results collectively support H5, confirming that mindful technology use and techno-boundary crafting constitute a significant behavioral pathway through which HRDRM generates sustainable work–life balance—a pathway that complements rather than replaces the direct structural H4 effect, thereby establishing partial sequential mediation.

**Table 13:** Sequential mediation results (bias-corrected bootstrapping, 5,000 resamples).

Pathway	Indirect *β*	LLCI	ULCI	Result
HRDRM → MTU → TBC → SWLB	0.039	0.020	0.069	Supported

Indirect *β* represents the standardized sequential indirect effect estimated using bias-corrected bootstrapping with 5,000 resamples. LLCI = lower limit confidence interval; ULCI = upper limit confidence interval.

## 6 Discussion

### 6.1 Overall pattern: a partial sequential mediation architecture

This study advances a partial sequential mediation model in which HRDRM influences SWLB through two complementary pathways: a direct organizational pathway (H4) and an indirect sequential behavioral chain through MTU and TBC (H5). This dual-pathway architecture is a substantive theoretical contribution, demonstrating that HR digital risk governance generates sustainable balance not only through structural provision—reducing digital demands at the organizational level (H4)—but also through a theoretically ordered sequence of individual behavioral competencies that organizations cultivate but cannot directly install (H5). The confirmation of all five hypotheses across Saudi Arabia's IT, banking, and higher education sectors provides both theoretical precision and external validity to findings that extend and empirically specify prior conceptual models of digital work and work–life balance (2, 40).

### 6.2 HRDRM and mindful technology use (H1)

The significant positive effect of HRDRM on MTU (Std *β* = 0.382, *p* < 0.001) is consistent with Dual Process Theory's proposition that reducing the ambient cognitive demands of System 1 reactive technology behavior creates the psychological space in which System 2 deliberate self-regulation becomes organizationally accessible (11, 12). When HR digital risk frameworks establish clear digital availability norms, protect recovery time, and legitimize deliberate disconnection, employees gain both structural permission and cognitive bandwidth for intentional technology engagement—a pathway supported by evidence that organizational support for digital boundary regulation strengthens deliberate self-regulatory practice in technology-intensive work contexts (21, 22). That HRDRM explains 15.0% of variance in MTU (*R*^2^ = 0.150) reflects meaningful institutional influence while leaving substantial residual variance attributable to individual factors—trait mindfulness, self-regulatory capacity, and openness to deliberate behavioral change—that future research should examine as moderators of this organizational-to-individual pathway.

### 6.3 HRDRM and sustainable work–life balance: the direct organizational pathway (H4)

The significant direct effect of HRDRM on SWLB (Std *β* = 0.338, *p* < 0.001) establishes that organizational HR digital risk governance generates sustainable balance outcomes independently of the sequential behavioral chain. This finding aligns with Boundary Theory's contention that sustainable work–life balance depends not only on individual boundary management behaviors but on the organizational boundary conditions within which those behaviors operate: organizations that structurally govern digital permeability through availability norms, workload protections, and recovery mandates produce measurable balance improvements regardless of whether individual behavioral competencies are fully developed (15, 16). This is consistent with evidence that organizational work–life support exerts direct effects on balance sustainability in digital work contexts (10, 24). Practically, the H4 finding establishes that structural HR policy and behavioral competency development are complementary rather than substitutable mechanisms—an insight that single-pathway models obscure and that managers implementing HRDRM programs should not overlook.

### 6.4 Mindful technology use and techno-boundary crafting: the developmental gateway (H2)

The MTU → TBC path (Std *β* = 0.547, *p* < 0.001) is the model's strongest, and its magnitude carries substantive theoretical weight: it establishes mindful technology awareness as the primary theoretically sequential precursor to proactive boundary crafting—not a correlated parallel strategy, but a necessary cognitive and behavioral foundation. Grounded in Temporal Self-Regulation Theory, this finding is consistent with the proposition that prospective self-regulatory competence—the capacity to design boundary architectures that pre-resolve future regulatory demands—grows from sustained practice in present-focused, immediate self-regulation (13, 14). Employees who have cultivated deliberate, awareness-based technology engagement accumulate the self-knowledge, intentionality, and behavioral confidence that proactive boundary design requires, a theoretically ordered progression supported by evidence linking mindful technology practice to enhanced boundary management capacity in digital work settings (10, 24). The non-significant direct path from HRDRM to TBC (Std *β* = 0.036, *p* = 0.411) is theoretically clarifying: it confirms that organizations cannot build boundary crafting capacity through risk policy alone but must first cultivate mindful awareness as the necessary gateway through which organizational support translates into individual boundary architecture competence.

### 6.5 Techno-boundary crafting and sustainable work–life balance (H3)

The TBC → SWLB path (Std *β* = 0.262, *p* < 0.001) confirms proactive boundary crafting as the proximate behavioral mechanism through which durable work–life balance is generated in the sequential model. Consistent with Job Crafting Theory and Boundary Theory, sustainable balance in digital work environments is produced not by reactive boundary defense but by the deliberate, self-initiated redesign of digital connectivity conditions—a behavioral investment that converts ongoing self-regulatory effort into a structural arrangement that sustains balance without continuous executive control (17, 23). The TBC mean scores (2.74–2.95) among Saudi respondents reveal that this most proximate balance mechanism remains systematically underdeveloped in a workforce navigating digital transformation at a pace that has outrun employees' development of the individual competencies needed to manage it sustainably (7, 40)—a finding that simultaneously underscores the model's relevance and amplifies the urgency of investment in boundary crafting capability.

### 6.6 Sequential mediation and the dual-pathway model (H5)

The statistically significant sequential indirect effect of HRDRM on SWLB through MTU and TBC [*β* = 0.039, 95% CI (0.020, 0.069)] confirms that the behavioral self-regulation pathway constitutes a meaningful and distinct mechanism for generating sustainable balance, operating independently of and in addition to the direct H4 pathway. The partial mediation structure carries an important theoretical implication: it establishes that the behavioral pathway is neither redundant with nor a substitute for structural provision, but the mechanism through which organizational policy is translated into internalized, self-sustaining individual competence. This distinction matters practically because balance generated through the H5 pathway—rooted in employees' own cultivated mindful awareness and boundary architecture—is sustainable even as organizational policy contexts shift, whereas the direct H4 pathway generates balance contingent on continued structural provision. Together, the two pathways provide a more complete account of how HRDRM shapes SWLB than either single-mechanism model could offer (9, 37).

### 6.7 Contextual contribution: Saudi Arabia's digitalizing knowledge economy

The Saudi Arabian context adds both interpretive depth and strategic urgency to the model's findings. The below-midpoint MTU and TBC means—reflecting a workforce that is highly digitally connected but not yet equipped with the self-regulatory competencies to manage that connectivity sustainably—mirror a broader pattern documented in rapidly digitalizing middle-income economies where technology adoption has outpaced behavioral infrastructure development (3, 8). Vision 2030′s human capital agenda positions knowledge workers in IT, financial services, and higher education as the primary engines of economic diversification; the present findings suggest that realizing this ambition requires investment in behavioral competency development—mindful technology engagement and proactive boundary crafting—as organizational infrastructure alongside the structural HR policy frameworks that have historically defined digital work governance in the Kingdom (7).

Although the proposed mediation model reflects a theoretically ordered sequence derived from established self-regulation theories, the present cross-sectional design does not permit direct observation of developmental change over time. Accordingly, the findings should be interpreted as supporting a theoretically specified sequential process rather than demonstrating an empirically observed developmental progression. Longitudinal or experience-sampling studies would be valuable for examining whether these proposed processes unfold temporally as theorized.

## 7 Practical and theoretical implications

### 7.1 Practical implications

The dual-pathway model of the study has a clear organizational message: structural policy and behavioral competence development are not options—they are consecutive necessity. The organizations which invest only on structural HRDRM processes get meaningful but partial results. Policies such as right-to-disconnect (prohibiting after-hours messaging), digital recovery protocols (requiring screen-free recovery windows), and recovery-time norms in team working agreements, all reduce digital boundary violations at source and lead to direct balancing improvements (H4). However, even good structural measures are not necessarily conducive to creating the individual attentive awareness and proactive boundary-crafting skill through which employees independently keep balance over time (H5). Structural protection sets the environment; behavioral self-regulation builds the capacity. The non-significant HRDRM → TBC route indicates exactly where the behavioral investment is most needed: MTU is the important sequential self-regulatory gateway. Since boundary-crafting capacity is cultivated by mindful awareness, and HRDRM directly cultivates awareness, the key programmatic investment is in mindful technology workshops. Such should be based on intentional self-regulatory practice, not generic wellness messaging: exercises in organized intentional gadget use, real-time attention monitoring, and purposeful disconnecting (19, 20). Importantly, these activities should not be done in isolation. Onboarding methods must establish mindful technological norms from the outset, positioning purposeful digital self-regulation as a professional expectation, not a personal coping mechanism. When leaders publicly model good digital etiquette—disconnecting, maintaining response-time limitations, and avoiding after-hours communication—it establishes the cultural permission needed for policy norms to become more than aspirational. For Saudi organizations navigating Vision 200's knowledge economy ambitions, these findings signal a capability gap that HR strategy must address explicitly. Mindful technology engagement and proactive boundary constructing are behavioral competences needed for sustainable digital work and should be recognized as strategic workforce capabilities, positioned alongside technical digital skills in national and organizational competency frameworks. HR leaders in Saudi Arabia's IT, financial services and higher education sectors should recognize that this combination addresses not only individual wellbeing, but the organizational productivity risks that unchecked digital burnout generates at scale (18, 25). They should design integrated HRDRM programs that combine structural protections with intentional behavioral self-regulation tracks.

### 7.2 Theoretical implications

This study makes three contributions to the digital work and HRM literatures. First, it introduces Human Resource Digital Risk Management as a theoretically coherent organizational-level antecedent—distinct from general HR support constructs—that systematically governs the psychosocial risks of digital work and translates organizational risk governance into individual behavioral outcomes. This positions HRDRM in an HRM paradigm focused on risk, which the discipline has not yet defined in the literature based on Armstrong and Taylor's (9) HR practice framework, but extended to the digital psychosocial risk domain. Second, the study contributes to the understanding of behavioral mechanisms that link organizational HR practices to work–life balance by establishing a sequential theoretically specified pathway—HRDRM → MTU → TBC → SWLB—grounded in Dual Process Theory, Temporal Self-Regulation Theory, Boundary Theory, and Job Crafting Theory.

Critically, it provides the first empirical evidence that Mindful Technology Use is a necessary theoretical precursor to Techno-Boundary Crafting rather than a parallel self-management strategy—a finding with direct implications for TSRT's theoretically sequential sequencing propositions and for boundary management research more broadly.

Third, the partial sequential mediation architecture—in which HRDRM influences SWLB through both a direct structural pathway and the sequential behavioral chain—challenges single-mechanism models of organizational support and wellbeing, demonstrating that organizational HR risk practices operate through complementary and theoretically distinct routes whose combined account is more complete than either pathway alone can offer. Conducted in Saudi Arabia's rapidly digitalizing knowledge economy, the study further extends digital work scholarship beyond its predominantly Western empirical base, demonstrating that theoretically sequential logic of the sequential model holds in a non-Western institutional context where digitalization has outpaced behavioral infrastructure development.

## 8 Limitations and future research

The cross-sectional design, while appropriate for testing the theoretically specified structural model, precludes causal inference and does not permit direct observation of the theoretically ordered timeline through which MTU builds into TBC capacity. Longitudinal designs tracking employees across HRDRM policy implementations or MTU training interventions would substantially strengthen causal interpretation and illuminate how quickly the sequential process unfolds in practice. The reliance on self-report data for all constructs introduces common method variance risk that procedural safeguards and Harman's single-factor test mitigate but cannot eliminate; future research incorporating behavioral technology use measures—app usage logs, connectivity records, experience sampling methodology—would provide a more robust multi-method assessment of MTU and TBC constructs in particular. Although the proposed mediation model reflects a theoretically ordered sequence derived from established self-regulation theories, the present cross-sectional design does not permit direct observation of developmental change over time. Longitudinal and experience-sampling studies would provide a valuable opportunity to examine whether these theoretically proposed processes unfold temporally as suggested.

The single-country, three-sector sample limits generalizability beyond Saudi Arabia's knowledge-work context. Comparative studies across Gulf Cooperation Council (GCC) countries and between Saudi Arabia and established digital work economies would test the cultural and institutional boundary conditions of the sequential mediation model and determine whether the HRDRM → MTU → TBC → SWLB theoretically specified pathway holds across regulatory, cultural, and digital maturity contexts. The model's explained variance in SWLB (*R*^2^ = 0.170) points toward unexplored moderators—leadership digital culture, job autonomy, and trait self-regulatory capacity—and additional mediators that may further specify the mechanisms linking boundary crafting to sustainable balance, offering a productive agenda for model extension in subsequent research.

Another limitation is the wording of some items of HRDRM measurements. The construct was developed to capture employees' holistic perspectives of corporate digital risk governance, hence some items cover closely related characteristics of digital work risks that often co-occur in practice. This approach does, however, reflect the integrated nature of organizational digital risk management. Future research may benefit from more granular measures that allow for the separate assessment of specific dimensions such as digital overload, techno-stress, connectivity pressure, recovery opportunities, and psychological wellbeing. Such an improvement would enhance precision of constructs and enable more extensive psychometric examination.

## 9 Conclusion

This study provides a theoretically grounded and empirically validated account of how organizational HR digital risk management generates sustainable work–life balance in digitally intensive work environments. The partial sequential mediation model demonstrates that HRDRM influences SWLB through two distinct and complementary mechanisms: a direct structural pathway in which organizational risk governance reduces boundary violations and improves balance at source, and a sequential behavioral pathway through which HRDRM cultivates mindful technology awareness, which in turn enables the proactive boundary crafting that sustains durable, individually owned balance. Grounded in Dual Process Theory, Temporal Self-Regulation Theory, Boundary Theory, and Job Crafting Theory, this dual-pathway model offers a more complete and actionable account of the HRDRM–SWLB relationship than prior single-mechanism frameworks have provided, and introduces HRDRM as a theoretically coherent organizational-level antecedent in the digital work and work–life balance literature.

For Saudi Arabia's rapidly digitalizing knowledge workforce, the findings carry immediate and nationally relevant implications: the path to sustainable balance in digital work runs not only through organizational policy but through the behavioral competencies that policy must cultivate—and organizations that invest in both structural risk governance and theoretically sequential behavioral infrastructure are best positioned to realize the human capital aspirations at the center of Vision 2030's economic transformation agenda (8). More broadly, the study contributes a model of organizational-to-individual behavioral self-regulation that travels beyond the Saudi context: wherever digital transformation outpaces individual adaptive capacity, the sequential development of mindful awareness and proactive boundary design represents the behavioral pathway through which sustainable work–life balance is made possible, not merely prescribed.

## Data Availability

The original contributions presented in the study are included in the article/supplementary material, further inquiries can be directed to the corresponding author.

## Appendix A

**Table A1:** Survey questionnaire.

Section/Construct	Code	Item/Category					
Demographic information							
Gender	—	□ Male □ Female					
Sector	—	□ IT & Technology Services □ Banking & Financial Services □ Higher Education/EdTech					
Organizational Tenure	—	□ 12–24 months □ 24–48 months □ More than 48 months					
**Human resource digital risk management (HRDRM)**			**1**	**2**	**3**	**4**	**5**
	HRDRM1	My organization actively identifies and addresses employee well-being risks associated with digitally intensive work.	□	□	□	□	□
	HRDRM2	HR policies in my organization help reduce digital overload, techno-stress, and excessive connectivity pressures.	□	□	□	□	□
	HRDRM3	My organization promotes healthy digital work practices that protect employees' recovery time and psychological well-being.	□	□	□	□	□
	HRDRM4	HR practices in my organization are regularly adapted to support employee well-being in evolving digital work environments.	□	□	□	□	□
Mindful technology use (MTU)							
	MTU1	I consciously decide when and how to use digital technologies during my workday rather than responding automatically or out of habit.	□	□	□	□	□
	MTU2	I am aware of how my technology use affects my concentration, stress levels, and psychological well-being.	□	□	□	□	□
	MTU3	I deliberately limit my engagement with digital devices during personal time to protect my rest and psychological recovery.	□	□	□	□	□
	MTU4	I engage with digital tools with a clear and intentional purpose, avoiding habitual or compulsive checking behaviors.	□	□	□	□	□
Techno-boundary crafting (TBC)							
	TBC1	I proactively set personal rules about when and how work-related technology can enter my personal time.	□	□	□	□	□
	TBC2	I intentionally shape the digital boundaries between my work and personal spaces to align with my preferences and well-being needs.	□	□	□	□	□
	TBC3	I actively modify how digital technologies connect me to work demands in order to protect my personal recovery time.	□	□	□	□	□
	TBC4	I craft my own digital boundaries rather than simply accepting organizational technology expectations.	□	□	□	□	□
Sustainable work–life balance (SWLB)							
	SWLB1	I am able to maintain a consistent and healthy balance between my work responsibilities and personal life over time.	□	□	□	□	□
	SWLB2	The boundaries between my work and personal life are sustainable and do not generate persistent or cumulative strain.	□	□	□	□	□
	SWLB3	My current digital work arrangements allow me to engage meaningfully in both professional and personal domains.	□	□	□	□	□
	SWLB4	I can sustain my work–life balance even when my work becomes more demanding or digitally intensive.	□	□	□	□	□

1 = Strongly Disagree; 2 = Disagree; 3 = Neutral; 4 = Agree; 5 = Strongly Agree.
